# *Coronamoeba villafranca* gen. nov. sp. nov. (Amoebozoa, Dermamoebida) challenges the correlation of morphology and phylogeny in Amoebozoa

**DOI:** 10.1038/s41598-022-16721-2

**Published:** 2022-07-22

**Authors:** Alexander Kudryavtsev, Fyodor Voytinsky, Ekaterina Volkova

**Affiliations:** 1 Laboratory of Cellular and Molecular Protistology, Zoological Institute, Russian Academy of Sciences, 199034 Saint-Petersburg, Russia; 2 Department of Invertebrate Zoology, Faculty of Biology, Saint-Petersburg State University, 199034 Saint-Petersburg, Russia

**Keywords:** Phylogenetics, Taxonomy, Microbiology, Biodiversity

## Abstract

*Coronamoeba villafranca* gen. nov. sp. nov. is a small amoeba isolated from the surface planktonic biotope in the Bay of Villefranche (Mediterranean Sea). It has a confusing set of morphological and molecular characters. Its locomotive form is subcylindrical and monopodial with monoaxial cytoplasmic flow and occasional hyaline bulging at the anterior edge (a monotactic morphotype). Based on this set of characters, this amoeba is most similar to members of the genus *Nolandella* (Tubulinea, Euamoebida). However, molecular phylogenetic analysis based on only the small subunit ribosomal RNA (SSU rRNA) gene and on two concatenated markers (SSU rRNA gene and actin) robustly places this species in the Discosea, specifically, in a clade with *Dermamoeba* and *Paradermamoeba* (Dermamoebida) as the closest described relatives, and several SSU rRNA clones from environmental DNA. A unique glycocalyx of the studied amoeba consisting of complex separate units with pentameric symmetry may be considered a unifying character of this species with other dermamoebids. The monotactic morphotype demonstrated by these amoebae primarily occurs in Tubulinea but was recently confirmed in other clades of Amoebozoa (e.g. Dactylopodida and Variosea). This morphotype may be the plesiomorphic mode of cell organization in Amoebozoa that might have evolved in the last amoebozoan common ancestor (LACA) and conserved in several lineages of this group. It may reflect basic characteristics of the cytoskeletal structure and functions in Amoebozoa.

## Introduction

The amoeboid organization is one of the basic cell types that evolved independently in many lineages of eukaryotes^1^. Amoeboid cells in different major clades differ in the types and modes of activities of pseudopodia, temporary mobile projections of the cytoplasm controlled by the cytoskeleton (see^1,2^ for explanations of the basic terms). In some of the major eukaryotic clades, amoeboid cells are the main form for most species. One of these clades is Amoebozoa that comprise mainly amoebae with lobose pseudopodia, i.e. broad, blunt cytoplasmic projections consisting of hyaloplasm (optically transparent cytoplasm) and granuloplasm (part of the cytoplasm containing visible organelles and inclusions^2^). The modern classification system of Amoebozoa has been continuously developing from the study by A. A. Schaeffer who was the first to introduce a set of verifiable and reproducible characters into the study of amoebae^3^. One of the key taxonomic characters is the shape and structure of the locomotive form, a term understood as an amoeba cell in the process of stable and directional movement over the substratum. The size and morphological characters typical of this form (i.e. shape when viewed from above, shape in cross section, arrangement of hyaloplasm and granuloplasm, structures produced at the posterior end of the cell—a uroid) are characteristic for the taxa and allow identification and classification. Other characters include the shape of the floating (free-swimming) form, the size and structure of the nucleus, and certain ultrastructural characters (in particular, the ultrastructure of the cell coat), see^3–5^. The accumulation of knowledge about the diversity of locomotive forms led to the publication of the morphotype system, i.e., the designation of the categories of locomotive forms unified by similar characters under certain names^6,7^ to facilitate morphological identification. Application of the locomotive form and the mode of movement in the higher-level classification of lobose amoebae was attempted by Bovee and his collaborators^8–12^. It was not well accepted before the beginning of the molecular systematics era when it turned out that the main clades of Amoebozoa may be characterized morphologically based on the pattern of movement and pseudopodial formation^13,14^. The clade that immediately started to appear in all molecular phylogenetic trees was Tubulinea Smirnov et al., 2005, comprising amoebae primarily characterized by “producing tubular, subcylindrical pseudopodia or capable of altering the locomotive form from a flattened, expanded one to a subcylindrical one. Monoaxial flow of the cytoplasm in every pseudopodium or in the entire cell”^14^, p. 138. This clade of Amoebozoa has been retrieved since the first single-gene molecular phylogenetic works with a reasonable dataset size^14–17^ to multigene phylogenetic studies^18–22^. The cells’ ability to adopt a cylindrical shape or produce cylindrical projections with monoaxial cytoplasmic flow is considered as a synapomorphy of Tubulinea, and it is broadly assumed that this set of morphotypes does not occur in the other clades of amoebae. However, several recent studies revealed notable exceptions. For example, Van Wichelen et al. described *Schoutedamoeba minuta* Van Wichelen and Vanormelingen, 2016—a new genus and species of monopodial amoebae below 20 µm in size revealed as planktonic grazers in cyanobacterial blooms^23^. Contrary to their morphology, these amoebae seem to be related to Variosea based on the analysis of the small-subunit ribosomal RNA (SSU rRNA) gene sequence. Another example is a recently reinvestigated *Janickina pigmentifera* (Grassi, 1881) Chatton, 1953, a parasite of chaetognaths (arrow-worms). This amoeba contains a cytoplasmic symbiont *Perkinsela amoebae* (Hollande, 1980) that belongs to Kinetoplastida. This was the reason why this species was long included in the genus *Paramoeba*^24^ that now belongs to Dactylopodida, although it adopts a cylindrical and monopodial locomotive form that is extremely different from other paramoebids. Therefore, this species was transferred to a separate genus *Janickina* Chatton, 1953. Yet, a recent molecular investigation has shown that these amoebae indeed belong to Dactylopodida and branch with a clade comprising species of *Paramoeba* and *Neoparamoeba*, in spite of the differences in their locomotive morphology^25^. On a eukaryote-wide scale, it should be noted that a superficially similar morphotype is demonstrated by the amoeboid cells of Heterolobosea (Discoba). The shape of these cells is that of cylindrical monopodial amoebae, but one of their significant differences from monopodial members of Amoebozoa is predominantly eruptive mode of cytoplasmic flow^26^.

Increasing evidence suggests that the morphotypes considered typical for Tubulinea are not restricted to this clade, as there are at least two examples of their emergence in the other clades of Amoebozoa. Here, we report the third case of an amoeba showing the tubulinean morphological characters of the locomotive form, but branching outside the Tubulinea, and discuss the evolutionary implications of this finding.

## Results

### Amoeba cultivation and morphology

**Figure 1:**
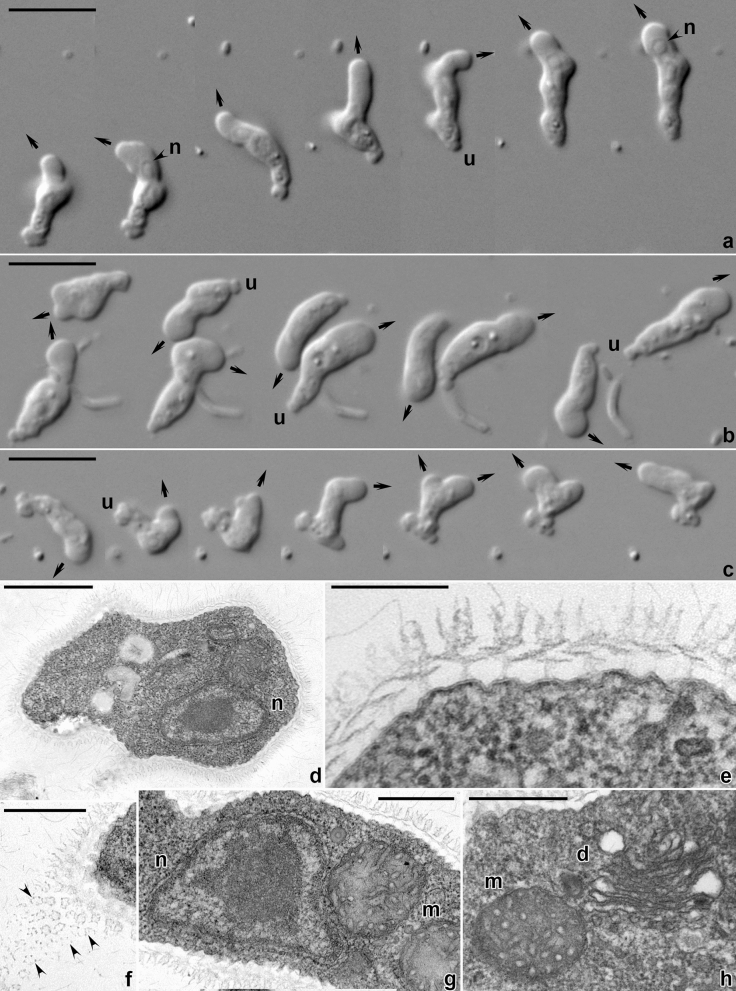
Morphology of *Coronamoeba villafranca* gen. nov. sp. nov. Light (**a**–**c**) and transmission electron microscopic (TEM) images (**d**–**h**). (**a**–**c**) Time-lapse sequences of the locomotion of amoebae (**a**,**c** show movement of one cell each; **b** simultaneous movement of two cells), DIC. Arrows indicate direction of advancement of the anterior end. (**d**) Low magnification TEM image of the whole cell. (**e**) Cell coat in a vertical section. (**f**) Cell coat elements in a tangential section (arrowheads). (**g**) Nucleus and a mitochondrion. (**h**) Dictyosome and a mitochondrion. *d* dictyosome, *m* mitochondria, *n* nucleus, *u* uroid. Scale bar 10 µm in (**a**–**c**), 1 µm in (**d**), 0.5 µm in (**f**–**h**), 0.2 µm in (**e**).

**Figure 2:**
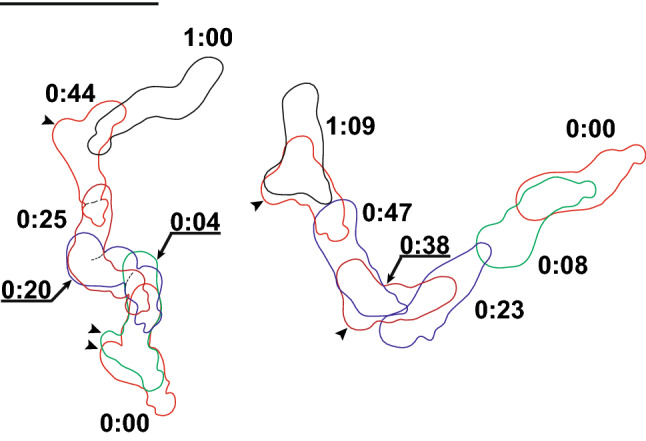
Kinograms of the locomotion of two cells on the glass substrate based on a video record. Time stamps are in minutes:seconds. Arrowheads indicate anterior bulges of the hyaloplasm. Scale bar = 10 µm.

Amoebae were not seen in the freshly inoculated sample, but notable after incubation of the sampled material in seawater with wheat grains. The studied strain could be easily purified and maintained under the culture conditions described. During locomotion either in Petri dishes on plastic or on the glass surface on slides, amoebae adopted a monopodial subcylindrical shape, usually tapering toward the posterior end (Figs. 1a,b, 2; Supplementary Video S1). The anterior end was blunt and semicircular; anterior hyaline cap occupied up to a quarter of the cell length. The posterior end (uroid) frequently produced a spherical bulb separated from the rest of the cytoplasm with a shallow constriction (Fig. 1a,b). In some cells additional bulges were produced at the uroid giving a somewhat morulate appearance (Figs. 1c, 2). Sometimes the uroid was blunt or tapering. The cell advancement was usually accomplished by a bulge-like expansion of an anterior hyaline cap past a constriction of the cell that remained stationary with respect to the substratum (Fig. 2; Supplementary Video S1). At some point the anterior end attached to the substratum, and the new leading edge started to bulge immediately on the anterior end of the cell, often in a direction different from the previous one (Figs. 1a–c, 2). Consequently, the cell usually moved in a sinusoidal path with a steady flow of the granuloplasm and occasional eruptive bulging at the anterior end (Fig. 2; Supplementary Video S1). Stationary and floating amoebae were usually irregularly rounded with the smooth surface.

The amoebae were uninucleate. The nucleus in the locomotive form was located in the anterior part, close to the border between hyaloplasm and granuloplasm. The shape of the nucleus was rounded or ovoid, with the large central nucleolus (Fig. 1a,b). The granuloplasm usually contained small spherical granules and several food vacuoles with bacteria. Cysts were never observed in our cultures.

**Figure 3:**
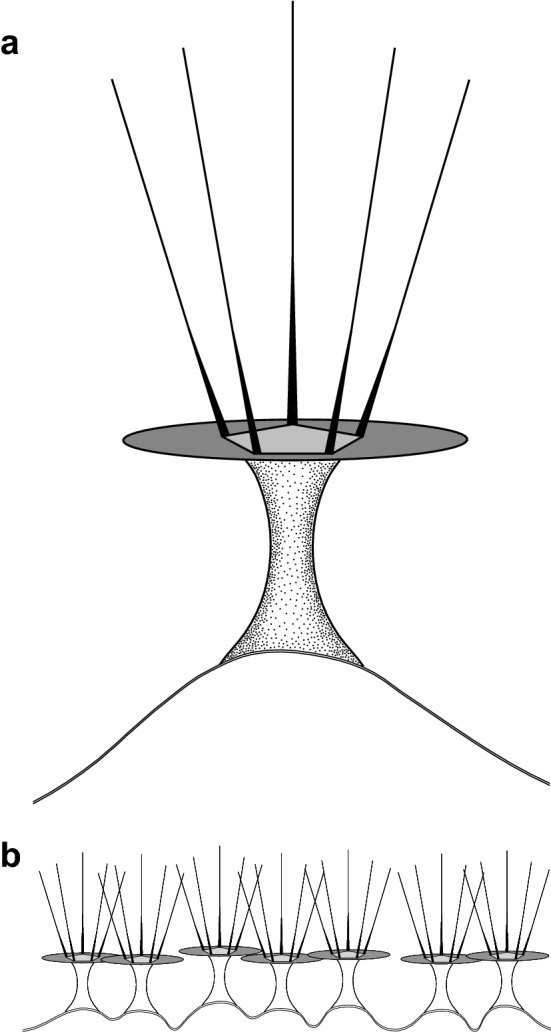
Diagram representing the reconstruction of the cell coat in *Coronamoeba villafranca* gen. nov. sp. nov. (**a**) Individual cell coat unit on the plasma membrane. (**b**) Scheme of the location of the cell coat units on the plasma membrane surface.

The transmission electron microscopic study revealed a cell coat over the plasma membrane that consisted of complexly structured units (Fig. 1d–f, 3). The plasma membrane demonstrated shallow bumps, on top of which the cell coat units were located (Fig. 1e). Units of the cell coat consisted of tower-shaped basal elements, on top of which flat, overlapping disc-like structures were located (Figs. 1e, 3). The disc-like structures were topped by the rising crown-like structures of pentameric symmetry (Fig. 1e,f). The height of the basal units was 22–72 nm (average 39.6 nm); height of the pentameric crown-like top structures, 69–134 nm (average 95.2 nm), diameter of the pentagonal structures was 56–81 nm (average 68 nm) (n = 73, 68, and 16, respectively). The nucleus in sections was irregularly rounded, with the central nucleolus and a layer of peripheral electron-dense chromatin under the nuclear envelope (Fig. 1d,g). The mitochondria were rounded or ovoid in sections and showed branching tubular cristae (Fig. 1g,h). Occasionally, cisternae of rough endoplasmic reticulum were seen adjacent to mitochondria. The Golgi dictyosomes consisted of 5–7 cisternae (Fig. 1h).

### Molecular phylogenetic relationships

Two DNA samples purified independently from sequential passages of the clonal culture yielded the same amplification product for the partial SSU rRNA gene. The maximal length of the amplified fragment was 1825 base pairs and differences between separate molecular clones of the same amplicon were 0–0.50% (average 0.30%) of the nucleotide positions excluding primer sites. The G + C content of the longest sequence was 46.8%, which corresponded to the average value among sequenced clones. BLAST search in the NCBI database always yielded the same result: the sequence obtained showed a 97% identity to an uncultured eukaryote sequence IAFDv47 (AY835690) from the marine methanol-fed fluidized denitrification system^28^. Other hits included marine uncultured eukaryotes and random organisms from different eukaryotic clades, however, the identity was always 88% and lower. The total length of the actin amplicon was 796 base pairs excluding primers, and there were no indels in seven sequenced molecular clones. Based on the sequence differences between molecular clones, two putative actin paralogs could be detected, represented by a group of six molecular clones with 0–0.40% differences (average 0.26%; n = 15) and a single molecular clone that differed from the rest in 6.50–6.70% (average 6.58%; n = 6) of the nucleotide positions. This variability at the nucleotide level corresponded to the variability at the amino acid level. The translated amino acid sequences (standard translation table) of the molecular clones belonging to the first paralog differed from each other in 0–0.80% of the amino acid positions (average 0.40%; n = 15), while the second paralog differed from the rest of the clones in 6.80–7.20% of the amino acid positions (average 7.00%; n = 6).

**Figure 4:**
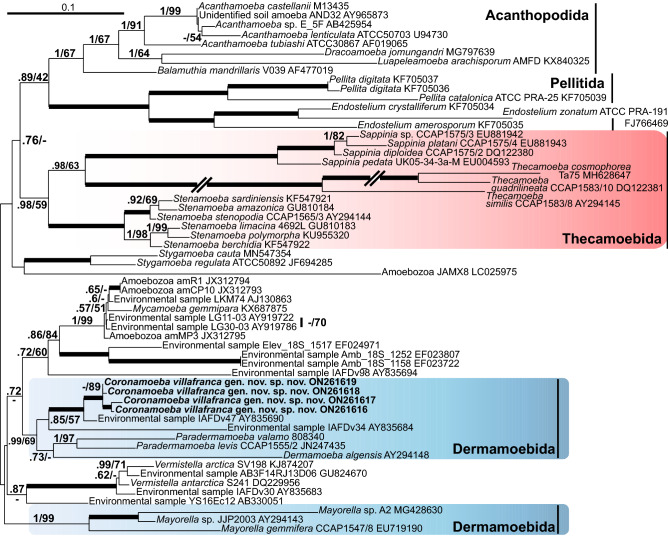
Part of the maximum likelihood phylogenetic tree based on the SSU rRNA gene analysis showing position of *Coronamoeba villafranca* gen. nov. sp. nov. (indicated in bold) among Dermamoebida and related clades. Numbers at nodes indicate posterior probabilities/bootstrap support values if above 0.5/50. Thick branches = 1/100. Scale bar 0.1 substitutions/site.

**Figure 5:**
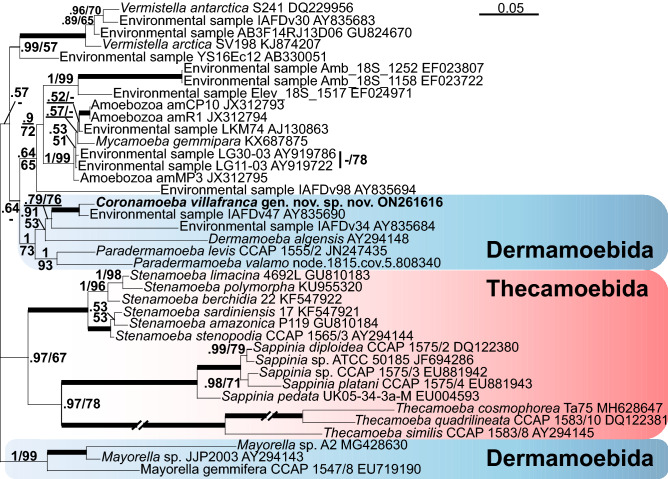
Part of the maximum likelihood phylogenetic tree based on the analysis of concatenated alignment of SSU rRNA and actin genes showing position of *Coronamoeba villafranca* gen. nov. sp. nov. (indicated in bold) among Dermamoebida and related clades. Numbers at nodes indicate posterior probabilities/bootstrap support values if above 0.5/50. Thick branches = 1/100. Scale bar 0.05 substitutions/site.

Phylogenetic analyses of 18S rRNA gene sequences always yielded the same result, regardless of the set of species in the alignment. The newly obtained sequences formed a 100% supported clade with an environmental sequence IAFDv47 (Fig. 4, Supplementary Fig. S1). This clade was sister to an environmental sequence IAFDv34, and the whole clade was sister to *Dermamoeba algensis*. The clade comprising new sequences and *Dermamoeba algensis* was sister to a clade of *Paradermamoeba* spp. (Fig. 4, Supplementary Fig. S1). The whole clade comprising the new species, *Dermamoeba*, and *Paradermamoeba* was sister to a clade comprising *Mycamoeba gemmipara* and a set of unnamed amoebozoans and environmental sequences related to it. This morphologically heterogeneous assemblage poorly supported at a higher level branched closely to Centramoebida, Thecamoebida, and Stygamoebida. Analyses of the concatenated dataset of 18S rRNA and actin genes yielded a similar result (Fig. 5, Supplementary Fig. S2). The topology and composition of the clade comprising a new strain was somewhat different from the one revealed by single-gene analyses based on 18S rRNA gene. In particular, in the former tree (Fig. 4), *Dermamoeba algensis* was sister to *Paradermamoeba*, but this position was only moderately supported with a posterior probability of 0.73 and not supported with the bootstrap value. In the latter tree (Fig. 5), *Dermamoeba* was sister of the clade comprising a new strain and environmental sequences (IAFDv47 and IAFDv34), and this position was supported with a posterior probability value of 0.91 and a bootstrap value of 53. The support for the clade comprising a new strain and Dermamoebida (*Paradermamoeba* and *Dermamoeba*) was 0.99/69 (Fig. 4) and 1/73 (Fig. 5), respectively.

### Reactions to salinity oscillations

**Figure 6:**
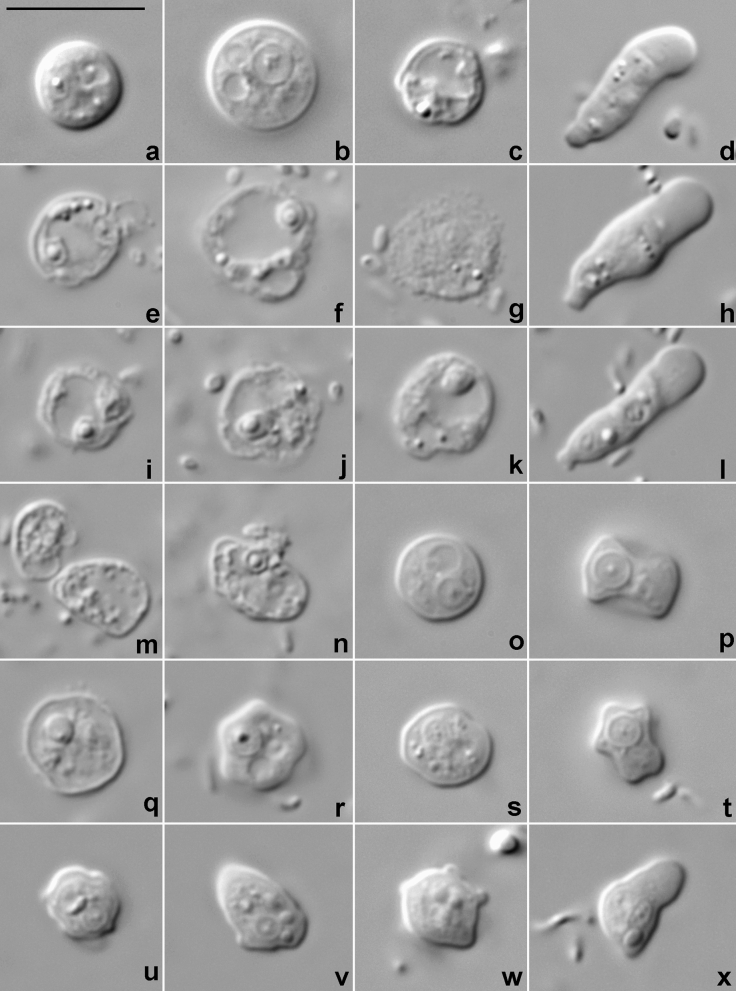
Light micrographs of *Coronamoeba villafranca* gen. nov. sp. nov. trophic cells on slides showing changes in amoeba morphology under the influence of the medium dilution. (**a**–**c**) Immediately after dilution to 0.3‰. (**d**) Locomotive form of a control cell in 40‰ immediately after placement on slide. (**e**–**g**) After 1 day of incubation in 0.3‰. (**h**) Locomotive form of a control cell in 40‰ after 1 day on slide. (**i**–**k**) After four days of incubation in 0.3‰. (**l**) Locomotive form of a control cell in 40‰ after 4 days on slide. (**m**,**n**) After 1 day of incubation in 1.25‰. (**o**,**p**) Immediately after dilution to 2.5‰. (**q**,**r**) After 1 day of incubation in 2.5‰. (**s**,**t**) After 3 days of incubation in 2.5‰. (**u**,**v**) Immediately after dilution to 5‰. (**w**,**x**) After 2 days of incubation at 5‰. Scale bar in a = 10 µm, valid for all micrographs.

Amoebae inoculated in experimental Petri dishes with different salinity values showed different responses. Immediately after inoculation, cells placed in the dishes with salinity value of 0.3‰ stopped all activities and took a rounded shape (Fig. 6a–c). After several minutes of observation, either movement or degradation of the cells were not detected. The amoebae inoculated in 18‰ medium were rounded for several minutes, but later resumed locomotion. No alterations in shape or behavior occurred in amoebae placed in 60‰. One day later, amoebae in 18‰ did not show any changes in activities compared to the control (40‰), in 60‰, some cells were rounded and adopted floating forms, but most of the cells remained active. The amoebae in 0.3‰ remained in a rounded shape and were motionless. One week later, the number of cells noticeably decreased in 0.3‰ medium, while no other changes occurred. Many amoebae in 0.3‰ medium presumably died, and their remnants remained in culture as irregularly shaped rounded envelopes with uneven surface (Fig. 6e–g,i–k). Amoebae in 18‰ and 60‰ were active and reproduced. Subsequent observations demonstrated a further decrease in the number and absence of activities in 0.3‰ and reproduction in 18‰ and 60‰. After 3 weeks of incubation, no amoebae remnants were seen in 0.3‰. Amoebae placed in the medium diluted to 1.25‰ showed the same changes in morphology as those in 0.3‰ (Fig. 6m,n). Amoebae in the medium diluted to 2.5‰ were spherical, irregularly rounded and motionless, without any changes during the observation period (Fig. 6o–t). Their cytoplasm was vacuolated, and numerous tiny granules performing fast, chaotic movements were seen inside the cells. Amoebae in the medium diluted to 5‰ were irregularly rounded or elongated (Fig. 6u–x), their shape sometimes resembled that of locomotive forms, and even a separation between hyaloplasm and granuloplasm was seen occasionally (Fig. 6x). Amoebae in the medium diluted to 10‰ ceased any movement immediately after medium substitution, but fully restored their morphology and activities in 1 day. The amoebae in control cultures in 40‰ treated and observed in the same way did not show any alterations in cell morphology or reproduction during the course of the experiment (Fig. 6d,h,l).

## Discussion

Identification of this amoeboid protist is difficult due to its confusing combination of morphological characters. The locomotive form of these amoebae is monopodial, subcylindrical (termed ‘limax’^29^), tapering towards the posterior end with a small bulbous uroid and occasional bulging of the hyaloplasm at the anterior end. It most closely resembles members of the genus *Nolandella* and, to some extent, *Vahlkampfia*. Although *Nolandella* was first established by Page in^30^, its first members were described several years earlier as members of the genus *Hartmannella*, *H*. *abertawensis* and *H. hibernica*^31^. The latter species was further designated as a type species of *Nolandella*, but the former one remained in *Hartmannella* until its 18S rRNA gene sequence became available^32^, but it was not until the revision by Smirnov et al. when the formal transfer was made based on the molecular data^33^. Many aspects of the locomotory behavior of *N*. *hibernica* are similar to those studied here. In particular, Page mentions that “These amoebae move somewhat like slow vahlkampfiids and have a corresponding form (Fig. 3). They put out hyaline hemispherical bulges rather slowly at the anterior end. To change direction greatly, they may put out a lateral pseudopodium, but otherwise the anterior end simply bulges out at an angle to the original course. They change shape and direction often, making measurement of locomotive rate difficult”^31^, p. 64. However, there are also some differences at the light microscopical level; in particular, Page describes that in *N*. *hibernica* some of the hyaloplasm runs back along the side of the cell as it moves and also that the knob-like uroid sometimes has trailing filaments, both features were not observed in the strain studied here. The ultrastructure of the strain studied rules out its identification as a vahlkampfiid or any other heterolobosean, as it shows mitochondria with branching tubular cristae, and pronounced dictyosomes^27^. The type of the cell coat that we demonstrated in the studied strain was never observed in amoeboid protists before. Definitely, the cell coat of *Nolandella* has a different structure, although some ultrastructural characters of this genus are similar to our strain: for example, a layer of electron-dense material under the nuclear envelope.

Hence, based on the morphological characteristics, this strain is most similar to *Nolandella* (Amoebozoa, Tubulinea) apart from its unique cell coat, but it shows completely different and unexpected molecular phylogenetic relationships. On the basis of the SSU rRNA gene analyses, this amoeba appears to be a member of Dermamoebida that is represented by *Dermamoeba* and *Paradermamoeba* in our trees. In contrast to the currently accepted phylogenetic hypotheses^1,20,33^, the genus *Mayorella* in our trees is not part of the Dermamoebida, but rather groups as sister to Dermamoebida + *Mycamoeba* + *Vermistella*, although the support for this grouping is low. The new strain forms a robust clade with members of Dermamoebida and two uncultured amoebozoans from the marine environment (IAFDv47 and IAFDv34). This result is reproduced with all tree reconstruction algorithms and datasets, and this clade constantly appears to be a sister to the lineage of *Mycamoeba gemmipara* and several unnamed amoebozoans (collectively designated as *Mycamoeba*^1^). This tree topology is unexpected, because it contradicts the locomotory pattern of the strain studied which, as discussed above, is characteristic of Tubulinea, while *Dermamoeba* and *Paradermamoeba* have flattened locomotive forms with polyaxial cytoplasmic flow and without pseudopodia^33–36^. However, the cell coat structure of the strain described here may be considered a shared character with dermamoebids. In particular, in *Paradermamoeba valamo*, the spiral glycostyles making up the main part of the thick glycocalyx terminate with straight funnel-shaped structures that sometimes seem to be pentagonal in cross-section; their length is ca. 120 nm, width ca. 60 nm, and electron-dense plates are reported between these structures and spiral glycostyles^35,36^. Vertical structures with unresolved details have also been reported in the distal part of the cell coat of *Dermamoeba granifera*^34^. Similar structures (also poorly resolved) were observed in some sections of *D*. *algensis*^37^. In the strain studied here, the distal part of the glycocalyx is composed of distinct pentameric crown-like structures that are located on top of the horizontal plates (Fig. 3). We can suggest that these distal structures are homologous to the terminal parts of the glycostyles in *Paradermamoeba* and probably palisade structures of *Dermamoeba*. The main part of the glycocalyx typical for the dermamoebids might have reduced considerably in this lineage, probably due to the small size of these amoebae. Therefore, we suggest the inclusion of the studied species into Dermamoebida with the establishment of a new genus and species within this clade. The data presented may also suggest a reconsideration of the inclusion of *Mayorella* into Dermamoebida that was based on the previous phylogenomic studies^20,21^. The datasets used in these works were typically limited to members of the genera *Dermamoeba*, *Paradermamoeba* and *Mayorella*, and did not include either *Mycamoeba* or the new lineage described here. However, a very similar tree topology was demonstrated by Cavalier-Smith et al.^19^ based on SSU rRNA gene analysis. We can hypothesize that the inclusion of more species in the phylogenomic trees would strengthen the current topology based only on the SSU rRNA gene dataset. An alternative option that has to be tested is the expansion of Dermamoebida to include current genera^1^ as well as *Mycamoeba* and *Vermistella* (Fig. 4). Phylogenomic data are highly desirable for the latter genera, as well as for the new species studied here.

The studied amoeba species shows a typical limax locomotive form of Tubulinea that is surprising given its evolutionary relationships, but contributes to the growing evidence that this morphotype may not be restricted to Tubulinea contrasting to the proposal by Tekle et al.^22^. Instead, it is present in a diverse set of lineages broadly scattered over the tree. Among them, *Entamoeba* spp. should be mentioned first (e.g.^38,39^). Furthermore, cylindrical monopodial locomotive forms were reported for the parasitic discosean genus *Janickina*^24^ which turned out to belong to Dactylopodida according to molecular data^25^, and the variosean genus *Schoutedamoeba* described relatively recently^23^. Moreover, on a broader scale, in the presence of certain eruptive flow on anterior end of the cell the amoeba studied resembles amoeboid members of the Heterolobosea^27^, a lineage distant from Amoebozoa or Amorphea in general^1^.

Based on the data presented, two hypotheses on the evolution of cellular morphotypes and modes of movement in Amoebozoa can be proposed. The first one implies that a monotactic morphotype characteristic primarily of Tubulinea was probably ancestral for the majority, if not all, deeply-branching phylogenetic lineages of Amoebozoa. This may reflect some fundamental features of the cytoskeleton and the mechanisms of movement in this supergroup. In this case, other morphotypes observed in different lineages of Amoebozoa might have evolved on the basis of the monotactic morphotype, with its conservation in some of the lineages. The second possible explanation is the convergent origin of this morphotype in different amoebozoan lineages. With the expansion of the biodiversity studies based on detailed analysis of morphological and molecular data, multiple cases of convergence have been recognized in the evolution of microorganisms, including even interdomain convergence (e.g., between bacteria and eukaryotic microbes; reviewed in^40^). According to the classification in^40^, the case of convergence demonstrated by the studied species falls under the “Category 1” (i.e., similar morphologies that evolve in genetically distant lineages). Based on the data available, we cannot choose at the moment, which of the proposed options is more probable. To clarify this, we need to understand cellular mechanisms that drive the formation of this morphotype, which remains a challenge. Based on studies on a few experimental systems, we can assume that the cytoskeletal mechanisms involved in this process are based on the interactions between the actomyosin system and accessory proteins (e.g.,^41^). However, the details of these systems are poorly studied for the variety of amoebozoan morphotypes and modes of movement. Understanding of these features for a broad range of morphotypes in Amoebozoa may lead to the clarification of the origin and evolutionary history of this supergroup in general.

Being an isolate from plankton, the studied strain represents a rare and probably significantly undersampled fraction of amoeboid protists inhabiting planktonic biotopes. In fact, it is most probably associated with a natural organic substrate, as it appeared in the inoculate of the material from a radiolarian colony. The amoebae are present in considerable numbers in planktonic biotopes, but are mostly associated with floating particles rather than free swimming in water^42^. It remains to be resolved whether there is a specific fauna of planktonic floc-associated amoebae that differs in its composition from the benthic one. The studied strain shows a good example of how difficult it is to distinguish species that belong there. In fact, small size of these amoebae may be considered the reason for their paucity in light-microscopic characters. A small cell volume simply does not allow the formation of diverse cytoplasmic projections and various shapes of a cell. This makes the distinction of these amoebae extremely difficult, as they all look superficially the same. A similar situation occurs in some other groups^43,44,45^. Reliable identification is only possible using electron microscopy and molecular data in this case. Therefore, if the hypothesis that the demonstrated limax shape of the locomotive form is basal for diverse lineages of the Amoebozoa is true, a broad taxonomic diversity can be hidden under seemingly identical shapes that requires careful attention to the observed diversity in the smallest Amoebozoa. The studied amoeba appears to be a truly marine species, as it did not show reproduction under culture conditions below 10‰. However, preliminary data on its ability to survive in small numbers under such conditions allow us to suggest its ability to resist at least short-term exposure to dilution of the biotope with fresh water, which may facilitate the distribution of this species.

## Taxonomic section

### Amoebozoa, Discosea

#### Dermamoebida, emend

Oblong, lancet-shaped, irregularly triangular or limax-like cells; with a smooth cell surface or with few wide ridges, never wrinkled; short, wide triangular pseudopodia and, in some, subpseudopodia of dactylopodial type; complexly structured cell coat, multilayered, consisting of tightly packed helical structures, or pentameric crown-like structures. *Dermamoeba*, *Paradermamoeba*, *Coronamoeba*, *Mayorella*.

#### *Coronamoeba* Kudryavtsev, Voytinsky and Volkova, 2022 gen. nov.

Amoebae with limax-like locomotive form, tapering posteriorly, with anterior hyaline cap. Occasional eruptive bulging at the anterior end and steady monoaxial flow of the granuloplasm. Mitochondrial cristae tubular, dictyosomes present. **Zoobank LSID:** urn:lsid:zoobank.org:act:B8368BF2-5A25-414D-905E-C3E8F3DE165E; **type species:** *Coronamoeba villafranca* Kudryavtsev, Voytinsky and Volkova, 2022; **etymology:** *Coronamoeba* (corona—“crown”, L.), refers to the cell coat structure that makes an amoeba look as if it is covered with small crowns.

#### *Coronamoeba villafranca* Kudryavtsev, Voytinsky and Volkova, 2022 sp. nov.

Limax-like amoebae, 6–11 μm long by 2–4 μm broad (average 9 by 3 μm), length:breadth ratio 2.25–5 (average 3), n = 59; single nucleus about 2 μm in diameter with a central nucleolus about 1 μm in diameter; locomotive form with occasional bulging at the anterior end, sometimes bulbous uroid; cell coat comprised by complex structures consisting of tower-shaped basal units and pentameric, crown-like top structures rising over horizontal overlapping discs; total thickness of the cell coat ca. 140–200 nm. **Zoobank LSID:** urn:lsid:zoobank.org:act:CA954460-EC9C-4CA6-AC22-6B38AA6FE8A8; **type location:** 43.683 N, 7.317 E, planktonic material, primarily Collodarian colony, from the surface water layer, Bay of Villefranche, Mediterranean, salinity ca. 38‰; **type material:** holotype consists of the type culture (accession No ZIN.2022.02, originally VF.R.S.12.9.2), Epon embedding for the electron microscopy (accession No F175) and DNA samples (accession Nos A633, A637) deposited with the culture collection of heterotrophic protists of the Zoological Institute, Russian Academy of Sciences; **reference sequence data**: GenBank accession numbers ON261612-ON261619 (SSU rRNA gene), ON260831-ON260837 (actin); **reference video record:** Supplementary Video S1, and https://youtu.be/cKbplXGmHJQ**etymology:** *villafranca* (an Italian equivalent to Villefranche), indicates isolation from the Bay of Villefranche.

## Material and methods

### Sampling and isolation

Amoebae were isolated from the plankton sample collected at 'point B' (43.683 N, 7.317 E), a monitoring point in the Bay of Villefranche, in September 2019 using a plankton net. The sampling procedures were described in^25^. A portion of sampled material was diluted with filter-sterilized natural seawater and aseptically sorted using a binocular microscope. Different macroorganisms and suspended particles were manually separated using sterile needles and inoculated into plastic Petri dishes filled with sterile seawater, with the addition of wheat grains. Inoculated samples were incubated for several weeks with monitoring for amoeba growth once every 3–4 days. The amoebae were detected using a Nikon TS2 inverted microscope with phase contrast and cloned by transferring separate cells into Petri dishes 40 mm in diameter with a glass capillary pipette. Filter-sterilized artificial seawater (40‰) supplemented with wheat grains was used as a culture medium.

### Light and electron microscopic study

The techniques for light microscopic investigation of living amoebae are described elsewhere, e.g.^46^. To measure sizes of the locomotive forms and nuclei, photographs of living amoebae were used. Measurements were made using Fiji^47^. For electron microscopy, amoebae in monoprotist culture were fixed in a Petri dish by replacement of the culture medium with the first portion of fixative. The fixation protocol consisted of a fixation with 2.5% (v/v) glutaraldehyde for 30 min followed by 1% (w/v) osmium tetroxide for 1 h (both steps performed at + 4 °C). Both fixatives were prepared with 0.05 M sodium cacodylate buffer supplemented with 40‰ artificial seawater. Further procedures and equipment were essentially the same as described earlier^46^.

### Salinity resistance experiments

Survival of amoebae in media with different salinities was evaluated in an acute experiment. The amoebae growing in 60 mm Petri dishes with the normal salinity medium (40‰) were inoculated in artificial seawater with alternate salinity values, namely 0.3‰, 18‰, and 60‰. For each experimental salinity two dishes were inoculated in parallel, two dishes with 40‰ seawater were used as a control. All dishes were supplemented with sterile wheat grains. The condition of cells in experimental and control dishes was recorded using an inverted microscope several hours after inoculation, on the next day, and during the subsequent month, once in 2–3 days. The experimental medium was replaced with 40‰ seawater in the dishes where no living amoebae were observed after one month of incubation. In the second series of experiments, the reactions of amoebae to different degree of medium dilution between 0.3 and 18‰ were evaluated. Dense cultures of amoebae grown in 40‰ medium were transferred to the media diluted to 0.3, 1.25, 2.5, 5, and 10‰ by pouring 40‰ medium and intense rinsing with the appropriately diluted medium. Control cultures were treated identically, but the medium used for substitution was not diluted. To record changes in cell morphology, amoebae from experimental cultures were placed on slides and photographed using an upright microscope Nikon Eclipse Ni-U with differential interference contrast (DIC). Control slides were also prepared from untreated cultures.

### Molecular phylogenetic study

Total DNA was isolated from the cultures using guanidine isothiocyanate method^48^. PCR amplification, cloning, and sequencing of SSU rRNA and actin genes were performed essentially as described in^49^. PCR primer pairs were A10s1 and s20R in combination with s6F and RibB^50^ for SSU rRNA gene, and by Yoon et al. for a portion of actin gene^51^. For the SSU rRNA gene, a total of seven clones were sequenced from two independently purified DNA samples. Seven clones of the partial actin gene were sequenced in both directions. For phylogenetic analysis, the sequences were aligned with our curated database of SSU rRNA gene sequences from amoebozoans, including several additional sequences from uncultured eukaryotes that produced significant hits in BLAST searches against the NCBI nr database when using our new sequences as queries^52^. Alignment was performed using MAFFT software^53^ with parameters set for accurate alignment (–localpair –maxiterate 1000). Poorly aligned positions were trimmed using trimAl^54^ set with − gt 0.5, − st 0.0005. The final alignment of the SSU rRNA gene used for tree reconstruction consisted of 223 sequences and 1637 nucleotide positions. Phylogenetic analyses of the SSU rRNA and actin genes were performed based on the concatenated dataset. For concatenation, the set of sequences was expanded to include as many species with both markers available as possible. The final concatenated alignment consisted of a total 257 SSU rRNA gene sequences, among which 77 species had actin sequences available. Concatenation was performed using Seaview v.5 software^55^. Phylogenetic analyses based on the alignment of the SSU rRNA gene were performed using the maximum likelihood algorithm with RAxML v.8.2.10^56^ and Bayesian analysis using MrBayes v.3.2.7^57^. RAxML used GTRGAMMA substitution model and 100 independent tree searches with random starting trees followed by bootstrapping with 1000 pseudoreplicates and mapping of bootstrap values on the best tree. Bayesian MCMC analysis was performed using GTR + G + I substitution model (eight rate categories), sampling was performed for 30,000,000 generations with a random starting tree and a burn-in of 0.25 of the samples. In the two-gene analyses the dataset was partitioned, and substitution model parameters for each partition were evaluated independently. The substitution model for amino acid alignment was LG^58^ with empirical amino acid frequencies. Bayesian analysis on the partitioned dataset was run for 100,000,000 generations until the chains converged. All analyses were performed using a computational cluster of the Zoological Institute of the Russian Academy of Sciences.

## Supplementary Information

Supplementary Video 1.

Supplementary Information 1.

## Data Availability

The datasets generated and analysed during the current study are available in the NCBI/GenBank, accession Nos ON261612-ON261619 and ON260831-ON260837.
