# Supplementary material for: Coronamoeba villafranca gen. nov. sp. nov. (Amoebozoa, Dermamoebida) challenges the correlation of morphology and phylogeny in Amoebozoa
Source: Sci Rep. 2022 Jul 22;12:12541. doi: 10.1038/s41598-022-16721-2 (PMC9307759; doi:10.1038/s41598-022-16721-2)
Supplement: Supplementary file 2 — Supplementary Information 1. [file 41598_2022_16721_MOESM2_ESM.docx]

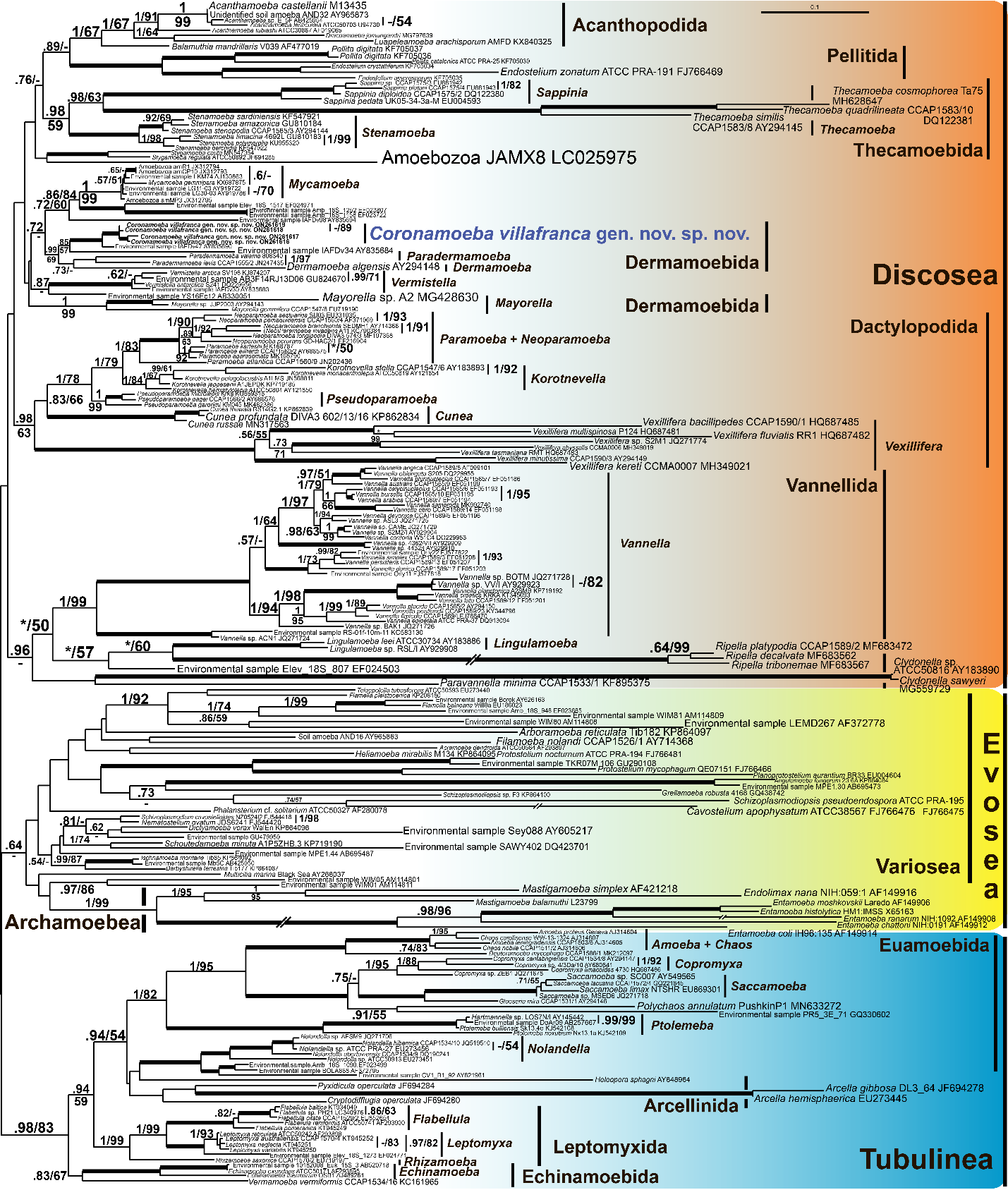


Supplementary Figure S1. Maximum likelihood phylogenetic tree of the main clades of Amoebozoa based on the SSU rRNA gene analysis showing position of *Coronamoeba villafranca* gen. nov. sp. nov. (indicated in bold). The tree is rooted between Discosea and other clades. Numbers at nodes indicate posterior probabilities/bootstrap support values if above 0.5/50. Thick branches = 1/100. Scale bar = 0.1 substitutions/site.


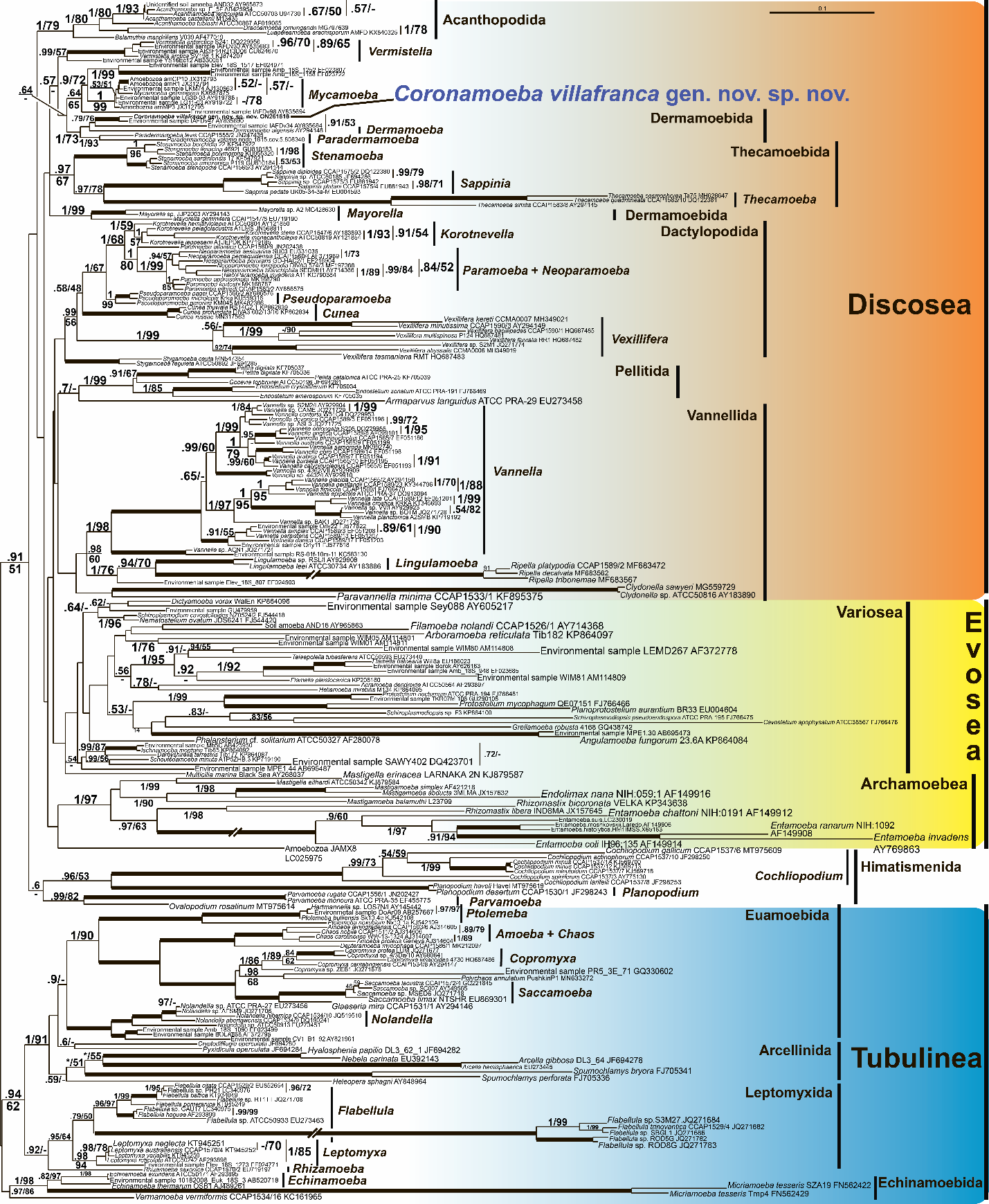


Supplementary Figure S2. Maximum likelihood phylogenetic tree of the main clades of Amoebozoa based on the analysis of concatenated alignment of SSU rRNA and actin genes showing position of *Coronamoeba villafranca* gen. nov. sp. nov. (indicated in bold). The tree is rooted between Discosea and other clades. Numbers at nodes indicate posterior probabilities/bootstrap support values if above 0.5/50. Thick branches = 1/100. Scale bar = 0.05 substitutions/site.

Supplementary Video S1. *Coronamoeba villafranca* gen. nov. sp. nov. Trophic cells during locomotion on glass surface in a temporary preparation. Differential interference contrast microscopy; scale bar = 10 µm.
